# Alternative Health and Conventional Medicine Discourse About Cancer on TikTok: Computer Vision Analysis of TikTok Videos

**DOI:** 10.2196/60283

**Published:** 2024-12-09

**Authors:** Roxana Mika Muenster, Kai Gangi, Drew Margolin

**Affiliations:** 1 Department of Communication Cornell University Ithaca, NY United States; 2 Department of Computer Science Cornell University Ithaca, NY United States

**Keywords:** misinformation, social media, TikTok, alternative health, cancer, computer vision

## Abstract

**Background:**

Health misinformation is abundant online and becoming an increasingly pressing concern for both oncology practitioners and patients with cancer. On social media platforms, including the popular audiovisual app TikTok, the flourishing alternative health industry is further contributing to the spread of misleading and often harmful information, endangering patients’ health and outcomes and sowing distrust of the medical community. The prevalence of false and potentially dangerous treatments on a platform that is used as a quasi–search engine by young people poses a serious risk to the health of patients with cancer.

**Objective:**

This study seeks to examine how cancer discourse on TikTok differs between alternative health and conventional medicine videos. It aims to look beyond mere facts and falsehoods that TikTok users may utter to understand the visual language and format used in the support of both misleading and truthful narratives, as well as other messages.

**Methods:**

Using computer vision analysis and subsequent qualitative close reading of 831 TikTok videos, this study examined how alternative health and conventional medicine videos on cancer differ with regard to the visual language used. Videos were examined for the length of time and prominence in which faces are displayed, as well as for the background setting, location, and dominant color scheme.

**Results:**

The results show that the alt-health and conventional health samples made different use of the audiovisual affordances of TikTok. First, videos from the alternative health sample were more likely to contain a single face that was prominently featured (making up at least 7.5% of the image) for a substantial period of time (35% of the shots), with these testimonial-style videos making up 28.5% (93/326) of the sample compared to 18.6% (94/505) of the conventional medicine sample. Alternative health videos predominantly featured cool tones (*P*<.001) and were significantly more likely to be filmed outdoors (*P*<.001), whereas conventional medicine videos were more likely to be shot indoors and feature warm tones such as red, orange, or yellow.

**Conclusions:**

The findings of this study contribute to an increased understanding of misinformation as not merely a matter of individual falsehoods but also a phenomenon whose effects might be transported through emotive as well as rational means. They also point to influencer practices and style being an important contributing factor in the declining health of the information environment around cancer and its treatment. The results suggest that public health efforts must extend beyond correcting false statements by injecting factual information into the online cancer discourse and look toward incorporating both visual and rational strategies.

## Introduction

### Overview

Misinformation has become a significant topic of concern not only in politics but increasingly also in nonpolitical spheres, especially in the realms of health and medicine [1-4]; for instance, members of the much-publicized bot and fake news farms in Macedonia, known for interfering in American elections, have also admitted to involvement in alternative health sites [5]. The COVID-19 pandemic not only resulted in a medical and public health crisis but also ushered in a public information crisis often referred to as an infodemic [6-10]. However, COVID-19 is not the only health issue about which falsehoods are prevalent on social media. Online conversations about cancer are similarly marred by misinformation [11]. Common claims suggest that standard treatments are neither safe nor effective and that supposedly corrupt pharmaceutical companies should not be trusted [12-14]. Some posit that the success of conspiracy theorists and those peddling falsehoods is a byproduct of the conservative nature of pronouncement: scientists’ tendency to speak on a subject only after they have sufficient certainty of the answer leaves many patients with unanswered questions. Those who are not governed by the norms and responsibility of academia are all too eager to try and fill this vacuum [14].

As with COVID-19, uncertainty and concomitant fear are prominent among patients with cancer [15-17]. With the urgency of a diagnosis often triggering information-seeking behavior, patients and carers search for help and support online, where they encounter not only unproven “alternative” treatments but at times also confounding and conflicting information from reliable sources [16-19]. The consequences of this confusion and misinformation can be dire because even a small delay in cancer treatment can have significant consequences for patient mortality [20-22]. Patients with cancer thus find themselves in an especially vulnerable position with regard to the misinformation present in online alternative health spaces [23].

Medical misinformation about cancer does not merely exist in response to patient demand; it has also been spurred on by the growing and flourishing industry of alternative health and wellness culture [24-26]. Content creators and social media platforms play a key role in facilitating the supply and encouraging the spread of health misinformation [4,27-29]. The rise of TikTok—the fastest-growing social media platform and one where falsehoods are plentiful—as a quasi-search engine for young adults has raised concerns among experts who worry that the platform will soon emerge as a new hotbed of misinformation [30-32].

In light of these developments, this study aims to examine cancer-related discourse and misinformation on TikTok. More specifically, we contrast the standard medicine and alternative health communities and demonstrate that not only do they differ in their narratives of illness and portrayal of the treatment of cancer, but they also use distinct visual-narrative languages when presenting these narratives. Using computer vision, we demonstrate that the 2 sides of the cancer community—one adhering to conventional medicine and another to alternative treatments—use differing visual rhetoric in their portrayal of healing and illness.

### Background

#### The Growth of Health Misinformation and the Alt-Health Industry

Research has uncovered 3 trends with respect to cancer-related misinformation on social media.

The first is that cancer misinformation is prevalent on the internet (as it is offline). In a study of popular social media platforms, Johnson et al [33] found that not only did a third of the articles about cancer contain misinformation, but many also included misinformation that could cause economic and physical harm to patients, such as encouraging a delay in treatment or touting supposed treatment with potentially toxic effects. This pattern of misinformation is consistent with other observations of cancer discourse on social media, where misinforming content often receives higher engagement than factual information [19,33-36].

A second, related concern is that this prevalence of misinformation distorts public understanding of cancer and its treatment. Results from the National Cancer Survey indicate that 40% of Americans believe that alternative medicine alone can treat cancer, and nearly half give credence to at least 1 medical conspiracy, such as the notion that the Food and Drug Administration is suppressing a cure for cancer, a view held by 37% of the respondents [37,38]. Even if individual falsehoods are not given credence, their profusion in online cancer discourse creates an aura of doubt and confusion, which is associated with reduced compliance with screening protocols [2,18,38]. The National Cancer Institute defines complementary and alternative medicine as “any medical and health care systems, practices, or products that are not thought of as standard medical care” [39]. Alternative health assumes a “holistic view of health [that] takes into account the ‘whole,’” contrasting it with the “mainstream” health system’s approach to illness [40]. In this way, the alternative medicine industry claims to meet patients’ needs in a way that conventional medicine cannot, providing an escape from “objectification, devaluation, and disempowerment” [41]. In actuality, many alternative health treatments for cancer have not been proven beneficial and safe, and some have even been shown to cause harm, including death, to patients [42,43].

Third, social media platforms have been instrumental in promoting the growing alt-health industry. Since its inception as a counterculture movement in California in the 1960s and 1970s, the so-called wellness culture has expanded into an intricate, interwoven system of goods and services, with the industry now estimated at US $1.5 trillion worldwide [29,44]. Between 1990 and 1997, researchers found a 47% increase in visits with natural health practitioners, while today between 20% and 80% of patients with cancer use natural methods to supplement their conventional treatment [45-47]. Alt-health, here, is used to differentiate between the substantial number of patients who may be using an alternative health modality in addition to their conventional treatment, and those individuals who form part of a movement of online promotion of alternative health bound in misinformation, conspiracy, and ideology [29]. The industry’s reach now extends to the period before diagnosis: the National Cancer Survey found that individuals are increasingly taking supplements—produced by an unregulated and sometimes unsafe industry—in an attempt to prevent cancer [37,48]. There is also a distinct financial component to alt-health; for instance, for the unsafe and ineffective “Gerson therapy,” patients are charged multiple thousands of dollars per week [43,49]. In 2018, an article in *The BMJ* posed the question, “Is cancer fundraising fuelling quackery?” It noted that, in the United Kingdom alone, US $10 million had been raised on crowdfunding platforms for supposed alternative cancer treatments since 2012 [50]. Social media platforms also serve to amplify the alt-health industry in other ways: alternative cancer clinics are using these platforms to advertise their wares, increasing the visibility of their unproven approaches to treating cancer [51].

A further connection between the alt-health industry and misinformation is its skepticism toward the motives and methods of conventional science and medicine. Health misinformation and alt-health discourse are commonly shrouded in theories suggesting that the corrupt pharmaceutical industry is motivated not by patient care but by profit—or, in more extreme cases, by a supposed desire to inflict harm on patients [52,53]. Scholarship highlights the disparagement of “big pharma,” a conspiracy theory used to refer to “an abstract entity comprised of corporations, regulators, NGOs, politicians, and often physicians” perceived to be financially benefiting from patients who are sick and dying [54]. In this view, conventional treatments are seen as toxic agents harming patients instead of treating them [23]. Alt-health proponents thus privilege supposed “natural” treatments over synthetic, “chemical” medicines. Following the logic of this so-called natural fallacy, what is natural is by default good, while anything chemical must be bad [23,55]. What this means, practically, is that alt-health can be influential by both promoting specific, sometimes dangerous, practices and undermining public confidence in established, medically supported treatments and practices. In 2018, an editorial in *The Lancet Oncology* voiced concerns over patients’ reliance upon such unproven therapies, arguing that increased distrust of the medical system leaves room for the growth in popularity of alternative, supposed treatments [56].

#### Visual Communication via Video Platforms

Digital platforms have been instrumental in the spread of falsehoods and antiscience attitudes. This is in part due to their infrastructure, which privileges engagement and virality, as well as their ability to connect disparate users, allowing them to share bias-confirming evidence and emotional support [57,58]. The rising popularity of video platforms adds another reason for concern because many of their affordances are well suited to promote alternative health information. Specifically, audiovisual social media platforms give nonexpert content creators the ability to create and disseminate content that is effective at persuasion through processes that do not follow logical reasoning.

Scholars argue that 3 elements are important with regard to an audiovisual message’s persuasive effect: what is said, how it is said, and how it looks [59]. Audiences frequently draw inferences about the credibility of a message through peripheral processing based on nonverbal cues given off by the speaker as well as the features of their presentation, such as vocal tone and how they are dressed [60]. As video is a richer medium, it affords the presentation of more of these cues. Social media platforms such as TikTok provide the creators of alternative health videos with an even wider range of editing tools, independent of evidence that they can (or cannot) provide for their claims.

Research suggests that visually presented information can influence persuasion even without engaging with judgments of credibility. Work on narrative persuasion shows that the features of a message can change its persuasiveness and the way audiences engage with it [60]. Narrative persuasion is not logical in the sense that it reasons with evidence and propositions [61,62]. Instead, narrative can be persuasive through the immersion of the audience in a simulated world in which causal relations are learned and then enacted to predict behaviors. Hamby et al [63] found that narratives have a larger effect on behavioral intent than nonnarrative formats, with some scholars attributing this effect to the ability of narratives to transport experience, motives, and metaphors as opposed to privileging rationality [61,64]. The effect is through the processes of embodied, imaginative simulation and interactions with identification, mirroring, and transportation [62,65,66]. These are derived from a variety of visual factors such as eye contact, facial expressions, emotive voices, or shared social or ethnic backgrounds [60,66-69]. When individuals identify with the characters in a story, they are both more likely to feel transported (increasing the story’s impact) and more likely to believe that the causal structure of the world portrayed in the story applies to them.

The role of these embodied simulation processes should be particularly important for persuasion regarding the effect of health-oriented interventions (such as disease treatments). This is because these interventions claim to make changes to an individual’s *body* and its healthfulness*.* Implicit in these claims is the idea that the treatment will make a person *feel* differently. By the logic of narrative, visual persuasion, the portrayal of treatments that make the audience feel a change toward healthfulness will seem to be effective; that is, if an individual *feels* healthy or perceives the person in the video as healthy after watching the video, they should be inclined to believe that the treatment has a healthful effect. Consider 2 ways to claim that an alternative to chemotherapy is effective. One way is to make arguments, perhaps based on statistical or even anecdotal evidence, that suggest that the alternative works. This argument may be augmented by peripheral cues, such as an authoritative voice, that lend credibility to the claims. Another way is for a patient to tell the story of their journey using this alternative method and to demonstrate or portray this journey visually. They might show still photos or video footage documenting the sadness they felt when they received the diagnosis and the happiness and vitality they experienced after their treatment—similar to the narratives seen in prescription drug advertisements [70]. The video demonstrates the effect of the treatment’s impact on the way the body feels and looks, rather than arguing for the effectiveness of the treatment based on how the body works. Audiences learn that people who use this therapy go from feeling one way (sad and sick) to feeling another way (happy and healthful), thereby concluding that the therapy is effective.

Consistent with this rationale, narrative testimonials are common in many media formats, including social media videos, and have been found to be more effective than health communication strategies based on the communication of statistical evidence [71-74]. For instance, Morton [74] posits that the use of narrative testimonials may aid influencers in the creation of authenticity and, by extension, trustworthiness. Studies of alt-health influencers show that personal testimonials, anecdotes, and subjective experience take precedence over expert knowledge and opinion [28,29]. Wellman [28] describes the influence of these emotionally engaging personal stories to be akin to that of “a friend who knows what they’re talking about.” This trust is easily abused; for example, an Australian wellness influencer profited greatly by falsely claiming that she had used diets and lifestyle products to cure herself of brain cancer [28,75]. Alternative cancer treatment videos may thus differ from standard cancer treatment videos in ways that emotively and visually highlight the supposed efficacy of treatments, not by making explicit false claims but by portraying an understanding of the world that is favorable to the treatments they promote.

Unsurprisingly, social media platforms facilitate the engaging message format of videos. Writing about Instagram Stories, Bainotti et al [76] highlight the “proliferation of small stories,” where short-form video invites what they term “(micro)storytelling” or “micro-narrations.” It can be argued that TikTok’s infrastructure privileges narratives and personal and engaging storytelling over fact-based information sharing. One way it does so is through its timeline, the ForYouPage: in a world in which many short videos are consumed in a continuous stream, there can be an effect where the whole is greater than the sum of its individual parts. Examining social media narrative genres, researchers see patterns of “small stories,” and fragments of storytelling [77,78]. Online storytelling is subject to cotellership, where interactions between users and the continuous scroll of videos placing individual videos in context with one another lead to them being seen not as fragments but as a whole, bolstering the overall credibility of their narrative [77-79]. Small stories and micronarratives are thus individual visual artifacts that function as fragments of a greater narrative, which, even if appearing or being seen separately, trigger prior instances of the same or a similar narrative.

In this view, influencers within the alt-health space thus create “tiles” of micronarratives in the form of videos, testimonials, and personal storytelling, which then form part of a wider alt-health narrative, parsed together by individual viewers [79]. Examining social media content as individual fragments, especially when it is provided as part of the endless flow of TikTok’s ForYouPage, neglects the impact the fragments have as recognizable examples of a genre. Examining them as representatives of a greater narrative instead highlights the fact that each video enters into an interdiscursive narrative with other examples of the same genre: users are unlikely to encounter merely 1 video pertaining to cancer; instead, they will encounter numerous microcontent fragments at different times throughout the day and actively work to relate them to previously seen content [79].

Social media platforms do not just enable the sharing of visual content; they are also instrumental in their creation and editing. The rich multimodality and editorial affordances of audiovisual platforms, especially TikTok, mean that there is an abundance of choices and features for creators to include, further opening opportunities for new forms of visual storytelling [80]. The theory of affordances, coined by Gibson [81], refers to what an environment—ecological or otherwise—offers to those within it in terms of possible actions or uses. This theory has since been applied to the study of social media, among other fields, where it is used to understand how social media features shape the way a user engages with, and behaves in, their environments [82-85]. TikTok shares similarities with the internet and other social media platforms, such as offering users the ability to express themselves or be creative [86]. TikTok also boasts of other, more unique affordances relevant to visual storytelling, such as its replacement of a home or landing page with an algorithmically curated short-video feed for the user to watch without exerting great mental effort on curation, supporting the aforementioned micronarrative effect [87]. TikTok also differs from other social media platforms more focused on building networks in that it instead centers, and allows for, escapist entertainment, similar to television [86].

Another affordance offered by TikTok is an extensive array of options for creating and editing content, such as filters and stickers, as well as the ability to respond to other videos or comments with a video called stitching or dueting [87]. Drawing on the concept of mise-en-scène and visual genres in the analysis of these social media videos can highlight the constructedness of TikTok videos: creators are not merely haphazardly turning on their camera; they are also making choices about angles, backgrounds, props, setting, music, and text subtitles, all of which carry implicit meaning [59]. Prior research has examined the prevalence of certain features, such as color, in social media posts of the same genre [88]. While the exact impact of color tone is still unclear, studies have found cool tones to be associated with lower engagement [89] and fact-checking videos to be associated with warmer colors [88]. Outside of TikTok, the persuasive effect of faces and the long history of the use and efficacy of testimonials in advertising have been established in prior research, but their use in social media has been discussed to a lesser extent [29,72]. Previous scholarship has pointed to the effect of the presence of faces on engagement rates, and humans’ tendency to focus on the faces in an image has long been established [88,90-93]. It is not just the mere presence of faces but also the way in which they appear that has become of interest: research indicates that expressions of joy and surprise are positively correlated with greater attention and viewer retention [94].

TikTok is also increasingly relevant due to its widespread use, especially among young people, who often use it as a search engine, and because it ushered in a new type of shorter-form video content that has since been adopted by other platforms due to its success [32,95-97]. TikTok’s success and popularity, especially among young people, have altered the ways in which users experience social media [87]. Long known primarily as an app through which teenagers shared dance videos, the use of TikTok has since expanded to other groups—including political campaigns, which now use TikTok to reach young voters and difficult-to-reach age groups [98-100]. In doing so, the campaigns have also adapted to the norms of communication on TikTok, often sharing (or performing) personal moments and life content, and entertaining material, rather than focusing on communicating policies [101,102]. Others have found that political communication on TikTok is more participatory and collaborative than on other platforms [103]. These features of communication on TikTok have to do with its primary user base, the affordances it offers to them, and the norms that develop around their use [103].

For our analysis, these findings on visual analysis and narrative suggest that we should aim to analyze the visual language of the alt-health and conventional health discourse on TikTok as a whole, with individual videos as parts of a greater narrative, rather than separate fragments. Thus, in examining the public communication environment on TikTok, a representative of the increasingly audiovisual social media landscape, we focus our analysis on the way in which these narratives are represented through visual choices, as well as on whether specific features such as background, location, and color are associated with either side. In this way, a valuable overview is created of the cancer-related public communication environment on TikTok. On the basis of this review of extant literature, we propose the following research questions (RQs):

- RQ1: How do the visual narratives about cancer shared by the conventional medical community on TikTok differ from those shared by the alternative health community?
- RQ2: In what ways are these differences communicated through specific visual narrative features?

## Methods

### Inference Strategy

#### Overview

To answer our RQs, our analysis proceeded through 3 stages: observation, testing, and explanation. In stage 1 (observation), we used a pilot sample of videos to manually identify theoretically interesting patterns that computer vision could plausibly detect. We used this stage to generate hypotheses for the computer vision analysis. In stage 2 (testing), we tested these hypotheses on a larger sample of videos. In stage 3 (explanation), we conducted a qualitative analysis of the video contents in light of the hypotheses supported by the computer vision analysis.

#### Computer Vision

Images transport significant meaning, but their analysis is often cumbersome or limited due to the labor-intensive nature of manual coding. Computer vision analysis, an automated method of analyzing visual data, offers an ability to examine visual data at scale [104]. Common uses include image classification, where each image is assigned a label out of a set of categories (eg, correctly identifying an image of a bike); detecting the presence of an object within an image; or recognizing human faces and bodies [104]. Previous research by Lu and Shen [88] used computer vision analysis to examine the properties of fact-checking videos on Douyin, the Chinese version of TikTok, to help guide decision-making on content creation for fact checkers. In political science, Joo and Steinert-Threlkeld [104] introduced new methods to use computational methods not only for the analysis of written and spoken political data but also for the analysis of meaningful and influential imagery used in politics. As some features cannot be measured by the human eye alone and because manual measurement, where possible, often lacks scalability, computer vision analysis is a promising tool for examining and understanding the vast amounts of content and data uploaded to social media platforms every day.

#### Defining Cancer Communication

We identified public videos by searching TikTok for those containing the term *cancer*. However*,* an initial search using this term alone resulted in a large proportion of videos about either astronomy or cancer in pets. Thus, to operationalize references to cancer as a disease in humans, the search phrase was modified to include a second keyword to identify relevant videos, that is, those concerning a discussion of cancer treatment in humans, while excluding irrelevant results. These search terms, which were identified from prior iterations of the search, yielded search results explicitly discussing the disease and its treatment in humans, while minimizing false positives (Textbox 1).

**The search phrases used for the data collection process on TikTok.**

**Search phrases**

- Conventional medicine: cancer AND treatment OR cancer AND chemo
- Alt-health: cancer AND healing OR cancer AND naturalhealing OR cancer AND holistic OR cancer AND holistichealth OR cancer AND cure

Two pilot samples, consisting of 100 videos each from the conventional medicine and alt-health sides, were drawn in March 2023 by the second author. Search terms were tested for precision and proved satisfactory. Upon identification of the appropriate search strategy and the variables of interest, another set of samples, consisting of 2412 videos in total, was drawn in August 2023 for the testing phase. The samples were then processed to ensure relevance. First, duplicate videos were excluded. Second, videos whose captions did not include a combination of the term *cancer* and any of the other search terms were discarded from analysis. Next, videos were sorted into alt-health and conventional medicine categories based on the inclusion of the respective search terms in the video captions. Videos that used terms from both conventional medicine and alt-health categories (eg, *cancer, treatment,* and *cure*) were sorted into both categories because average users would feasibly encounter them with either of these search terms. Ultimately, of the original dataset of 2412 videos, we were left with 326 (13.52%) videos in the alt-health category and 505 (20.94%) videos in the conventional medicine category for analysis.

### Initial Manual Inspection of Videos

The lead author first examined the sampled videos in an exploratory process to identify meaningful features for our computer vision analysis. The pilot sample (200 videos) was screened to identify visual patterns that were (1) detectable by computer vision; and (2) theoretically meaningful as it pertains to the arguments on visual storytelling outlined previously. For example, some videos from the alt-health sample showed people chopping vegetables as part of their routine when preparing a home remedy for cancer—while this is theoretically interesting and did seem to appear frequently in only a certain type of video, training the machine to reliably identify this particular behavior would require identifying hundreds of training examples for different vegetables in different forms. Similarly, the computer might be able to detect a feature such as the wearing of face masks, which satisfies the first point, but this feature’s theoretical meaning (the second point) could be obscured by external factors. For example, patients seeking chemotherapy may be more likely to appear masked in videos simply because they are filming themselves in places where mask wearing was mandated, such as hospitals, rather than making a conscious decision to include face masks in their videos.

This exploratory process pointed to 4 ways of conducting analysis on the larger sample using computer vision: the presence and prominence of faces (“testimonials”), scene and nature, color choice, and emotion displays. The rationale for these is described in the following paragraphs. On the basis of the pilot, we developed hypotheses for what we would observe and test for in the larger sample.

The first tendency we observed was for videos to be in the form of a testimonial. We observed many videos that were (1) posted by an ordinary person (meaning neither a physician nor a celebrity) who is (2) personally affected and recounting their experience and (3) that show the person’s face prominently and for a prolonged period. This is consistent with the definition of a testimonial video [71,74]. We also observed that, consistent with the aforementioned theoretical rationale, alt-health videos featured a greater number of testimonials than conventional medicine videos. However, not all of these features can be detected by the computer. Therefore, we hypothesized that the computer vision implication of this style—the prolonged and prominent display of one person’s face, which we refer to as *testimonial presentation*—will be significantly associated with alt-health videos (H1).

Second, we observed tendencies with regard to the locations in which the videos were filmed and the presence of nature overall. Arguments for alternative medicine often rely on the so-called natural fallacy, which privileges what is perceived as “natural” over the “chemical” [23]. Consistent with this, we noted that many videos in the alt-health pilot sample featured natural elements such as trees or gardens. Conversely, we observed many features and props in the conventional medicine pilot sample that indicated a clinical or industrial environment. Although, as noted previously, it was not feasible to identify each object, we observed that simply identifying the setting of the video as “outdoors” (natural) versus “indoors” (not natural) provided an approximation of the natural versus not natural effect because most of the videos that showed “nature” did so in an outdoor setting, while those that did not used an indoor setting. Thus, we hypothesized that alt-health videos featured a greater number of outdoor settings than conventional medicine videos (H2).

The third tendency observed concerns the dominant color tone of the video. We noticed in our alt-health pilot sample a dominance of green and blue hues accompanying the outdoor and natural settings (greenery and the sky, respectively) and in our conventional health sample an often hospital-related presence of beige and gray. We expect this pattern to hold true in the larger sample (H3).

The fourth tendency relates to the emotions displayed by the people in the videos. Cancer and its treatment represent times of significant upheaval for patients, and in light of this and prior findings on human reaction and attention to emotions [94], we were interested in whether the range of emotions displayed differed between the conventional medicine and alt-health samples. We observed that the alt-health sample showed significantly more happy emotions, whereas the conventional medicine sample featured more expressions of sadness.

### Computer Vision Analysis

Computer vision tools were used to assess certain features at scale, as well as other features that the human eye alone cannot measure. The *face_recognition* Python package [105], which boasts 99.38% accuracy, was used to determine the number of faces present throughout the video, as well as their proportional size of the screen (H1). For a given video, the face percentage refers to the percentage of frames in a video in which ≥1 faces are detected. Face size is calculated by taking the average face size calculated for each frame that contains a face. We manually checked 50 (6%) of the 831 videos to determine the precision and recall of the analysis and found it satisfactory with an *F*_1_-score of 0.857.

Next, to assess the presence of nature and scene, we used the Massachusetts Institute of Technology Computer Science and Artificial Intelligence Laboratory’s computer vision, Places365 convolutional neural networks for scene classification [106]. This was used to analyze whether a video was recorded indoors or outdoors (H2). The convolutional neural network was trained on a Places2 database [107] of approximately 1.8 million images. It classifies frames as *indoor* or *outdoor* based on certain features, such as the presence of indoor lighting and man-made materials (eg, plastic) for indoor scenes, and the presence of a horizon, natural lighting, and natural fibers for outdoor scenes. While the program can provide additional scene specifications, such as categorizing the location further to, say, offices, courtyards, beer gardens, or dressing rooms, these capabilities were not used for this analysis due to a lack of theoretical reasoning or hypotheses for any of these locations. Precision and recall testing for scene classification proved satisfactory with an *F*_1_-score of 0.978. In conjunction with this, ColorKit [108] was used to determine color tone (H3). Color tone is measured directly by the computer rather than being an approximation of a human-observed ground truth; therefore, we did not evaluate precision and recall for this analysis.

Finally, facial expression recognition using computer vision analyses proved unsuccessful. Human inspection of the sample showed that while surprise was identified as the most common emotion by the machine, a large number of these assessments were inaccurate. We hypothesize that this might be due to the norms of speaking and engaging on social media platforms, which prioritize engaging narration, sensationalism, and emotiveness—all of which could contribute to facial expressions typically associated with surprise, a product of “social media speak,” so to speak. As a result, we dispensed with this measure for testing our hypotheses.

### Qualitative Investigation

Next, the lead author conducted an audiovisual close reading of a sample of the analyzed videos to identify themes and styles that highlight and explain the computational findings. This reading thus served to “identify, analyze, and report” the patterns in which the examined features and computational results manifest in the videos [109]. The qualitative investigation paid close attention to the themes or recurring styles in each set, how they differed between the 2 samples, and, crucially, how the computationally examined features appeared within them.

### Ethical Considerations

This study did not require ethics approval from an institutional review board because the data that were collected and analyzed are publicly available, posted to public TikTok accounts. Individual accounts are neither named nor described in detail. Any screenshots reproduced in this study from the publicly posted videos have been anonymized, including the users’ appearance and usernames. A reverse image search was conducted with the anonymized images to ensure that the original accounts would not be discoverable as a result of this study.

## Results

### Computer Vision Analysis

Testimonials were operationalized using a combination of face presence (ie, is a face present in the video in this frame?) and face size (ie, how large is the face?). Three-fold categorization yielded the results presented in Table 1.

H1 examines in which of the 2 samples face size and face presence are more prominent. Operationalizing face size and face presence as indicators of testimonial presentation, these videos were found to be more frequent in the alt-health sample than in the conventional medicine sample (H1) under the *loose* categorization (*P*<.001) but not under the *medium* and *strict* categorizations (medium: *P*=.19; strict: *P*=.13), although the direction is consistent with H1. This means that videos in the alt-health sample showed faces closer to the camera and for a longer period of time in the testimonial-style presentation that is commonly used in influencer content in video format and known to be persuasive in its narrative form in health communication [28,71,74].

H2 hypothesizes that outdoor settings will be more prominent in alt-health videos than in conventional medicine videos. The relationship between alt-health videos and outdoor scenery was found to be significant (*P*<.001). Videos uploaded by patients with cancer seeking conventional treatment were thus more often recorded indoors, whereas patients with cancer seeking and promoting supposed alternative treatments chose to record and upload videos shot outdoors or with greenery and nature present.

Finally, in examining color tone in our sample, we found that cool-toned videos were associated more with alt-health videos than with conventional medicine videos (overall: *P*<.001; first dominant color: *P*<.001, second dominant color: *P*<.001, and third dominant color: *P*<.001). Conventional medicine videos were therefore more likely to have dominant warm undertones such as red, orange, or yellow, whereas alt-health videos were more likely to be dominated by cool undertones such as blues and greens.

**Table 1:** Results for the facial recognition computer vision analyses of 2 samples from TikTok, one representing an alternative health approach to treating cancer and the other a conventional medicine approach.

	Conventional medicine (n=505), n (%)		Alt-health (n=326), n (%)	
	Testimonial presentation	Nontestimonial presentation	Testimonial presentation	Nontestimonial presentation
Loose (35% face presence, 7.5% face size)	94 (18.6)	411 (81.4)	93 (28.5)	233 (71.5)
Medium (40% face presence, 10% face size)	77 (15.2)	428 (84.8)	61 (18.7)	265 (81.3)
Strict (45% face presence, 12.5% face size)	47 (9.3)	458 (90.7)	41 (12.6)	285 (87.4)

### Qualitative Investigation

An audiovisual close reading of a sample of the analyzed videos demonstrated that the computational findings correspond to distinct styles and themes between the 2 categories.

**Figure 1:**
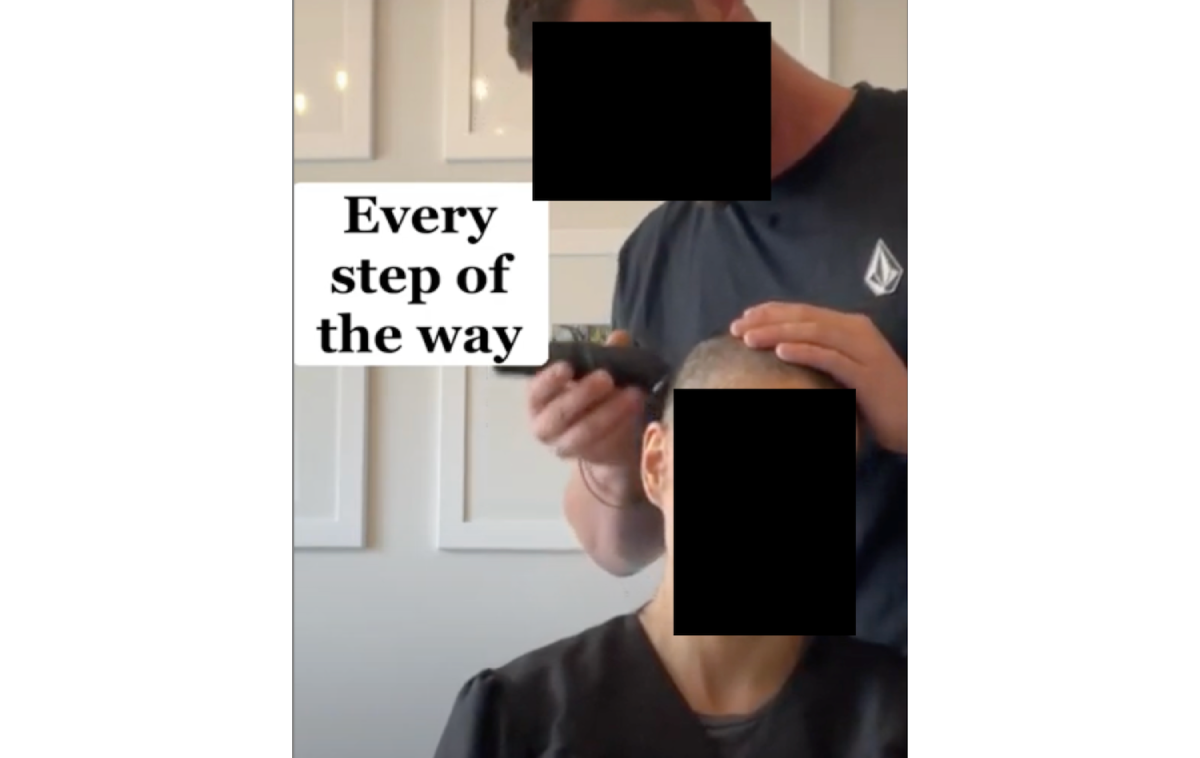
An example of the head-shaving genre in a TikTok video from the conventional health sample. The results from the computer vision analysis showed that videos, like this one, that feature or advocate for conventional medicine approaches to treating cancer are more likely to be filmed indoors than alternative health videos.

A distinct style of testimonial on the conventional medicine side shows the act of head shaving. Users frequently posted recordings of the shaving of their heads in a bathroom. The caption or in-video texts explain that this decision is in response to, or to preempt, the effects of chemotherapy, which often leads to drastic hair loss. Sometimes, the patient is seen alone, shaving their own head; more often, other people—family or friends—are seen helping the patient in the act of shaving. The mood is somber, as is the music choice, and sad expressions and tears are frequent throughout. The videos usually conclude with the user revealing their shaved head (Figure 1).

**Figure 2:**
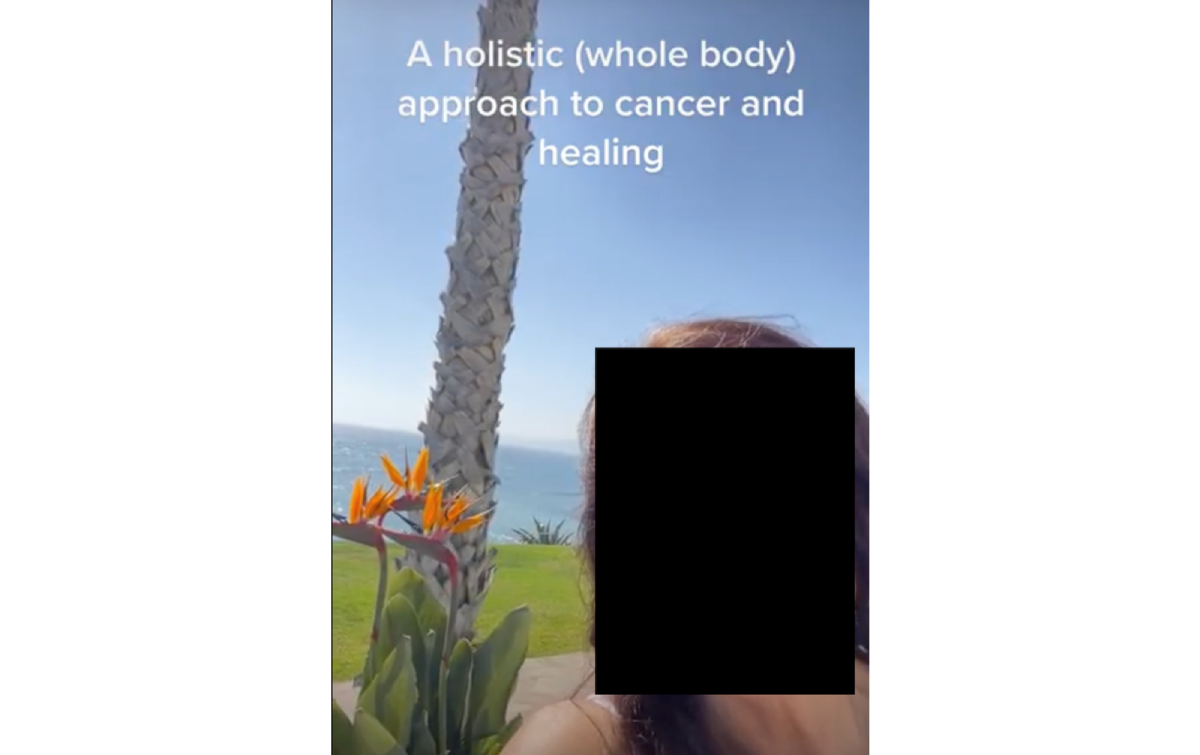
An example of the outdoors testimonial genre in a TikTok video from the alternative health sample. This screenshot exemplifies results from the computer vision analysis that showed that videos advocating for unproven alternative medicine approaches to treating cancer are more likely to be recorded outdoors and feature faces significantly (both in presentation duration and size).

Within the alt-health sample, “visuals of wellness” represents a common theme. Popular videos show the users, often women, in ways that do not make it apparent that they have been diagnosed with cancer: users are seen outdoors, with vibrant greenery, bright sunshine, and blue skies featuring prominently (Figure 2). The users are tanned, have long hair, and may be seen engaging in physical activity or preparing food. In the caption, the whole foods seen in the video—juicy fruits, greens, or spices—are claimed to represent much more than mere nutrition; they are purported to be the cure for the disease these users have, or had, been diagnosed with.

Visually, these videos exemplify the results of the computer vision analysis: the pattern of visual elements used within them mirrors itself across individual videos to form a genre. The visual choices of how the users in the 2 samples choose to portray their cancer journey, paint a drastically different picture: one highlights illness, while another obscures the signs of illness to highlight wellness; one shows the wide-reaching side effects of cancer treatment, while another shows a person who looks healthy after their cure; and one shows the patient with cancer visibly ill in windowless rooms or in hospitals, while another displays the patient apparently healthy and in nature.

These differences in visual genres, of course, are mirrored in the content of the sampled videos: conventional medicine videos usually highlight the pain, distress, and sadness of a cancer diagnosis and treatment cycle, often providing medical information about the exact type of cancer and its treatment. Alt-health videos, by contrast, contain misinformation casting doubt on the efficacy of chemotherapy or radiation (instead portraying them as poison), insinuating the existence of a cure, or presenting dangerous and ineffective ingredients or practices as safe and efficacious. Both video genres exemplify patient testimonials. In the conventional medicine sample, these patient testimonials differ from expert “lessons,” where physicians explain the science and mechanics of the disease. These videos feature medical personnel who convey their credentials not only through words and text (such as an account handle with “Dr. med.”) but also visually; by wearing scrubs, a laboratory coat, or a perfunctory stethoscope. In the alt-health sample, the “visuals of wellness” videos combine both patient and expert testimonials: users recount their experience as patients and build their expertise on the basis of this experience, proclaiming their cure, with their wellness projected as living proof of the treatment’s efficacy and thus their expertise.

## Discussion

### Computer Vision Analysis Findings

This study set out to examine how the visual language of videos about cancer on TikTok differs between those who support alternative health treatments and those who adhere to conventional medicine. The results show that videos advocating for unproven alternative health cures for cancer will more frequently do so in a testimonial-style video than those advocating for conventional medicine approaches, meaning that the alternative health videos will show users’ faces more prominently and for significant portions of the video. Similarly, support for natural health remedies is often underscored by natural scenery and backgrounds. When comparing the dominant color tones between the alt-health sample and the conventional medicine sample, the former tend toward cool tones and the latter toward warm tones.

In examining conversations about health, researchers have clearly established the prevalence of falsehoods and misleading claims and facts [51]. What our findings show is that there may be an additional layer to misinforming videos beyond the veracity of the claims made: there are substantial differences in the ways in which videos promoting alternative health “cures” for cancer attempt to persuade their audience visually. The alt-health videos we examined use visual social media to create a more transportive effect, one that fosters a personal connection with the audience, heightens the perceived authenticity of the user, and highlights the purported healthful life and path their chosen treatment affords. By contrast, the conventional medicine sample features videos that highlight the emotional distress of a diagnosis and the arduous process that treatment involves: videos displayed testimonial presentation to a lesser degree, showed a greater degree of sadness, and filmed themselves in hospital settings. It is possible that the patients with cancer in the conventional medicine sample did not feel the need to use persuasive video elements such as testimonial presentation because these videos often lacked the commercial impetus of alt-health videos or because they instead highlighted those aspects of their life that helped foster a connection to other patients with cancer by making their illness visible [110].

The 2 examples of common themes further exemplify the findings from the computational analysis: video recordings of patients shaving their head are usually shot from farther away than the personable close-ups in the alt-health sample, leading to the differences in face size and face presence between the 2 samples in what we label a testimonial presentation. Moreover, while the act of shaving might require a power outlet, thus making it more likely to take place indoors, other types of videos in the conventional health sample also did not feature outdoor backgrounds in the way that alt-health videos did. In addition, users could have chosen to share another type of video but instead adhered to a genre or style of video that is common within their community and puts them in communication with other members of this community.

Our findings indicate that there is little visual overlap between the alt-health group of patients with cancer and the conventional medicine group of patients with cancer on TikTok, mirroring the findings of Milani et al [111] that show that pro- and antivaccine users on Twitter (subsequently rebranded X) formed 2 separate, barely interacting networks. Some of the features, such as the role of testimonials, reflect a common genre of social media content that can be used by public health practitioners to build familiarity and credibility with potential viewers. Our findings also point to the necessity of considering the evolving nature of social media in health communication research and practice: viewing digital misinformation through the lens of the impact of a single message could leave us blind to the reality of social media consumption, which relies on small stories spread across fragments of videos and posts spreading the same visual narrative and adhering to features of the same genre [76,77].

The visual languages used by the users in the 2 samples paint 2 starkly different pictures of the journey and treatment of a patient with cancer. Users promoting alt-health on TikTok videos and images espouse purported natural cancer cures, claiming that these alternatives can cure cancer in days or weeks and without the uncomfortable side effects of conventional (actual) treatments. This portrayal presents them as a more attractive alternative to the arduous chemotherapy or radiation treatments depicted in the conventional medicine sample videos. These claims are then reinforced through visual narratives highlighting nature and natural objects, building an aspirational image often at play in the creator culture and constructing narratives of wellness: healthy, happy people enjoying life in nature. By contrast, the conventional medicine side builds and displays narratives of illness: users are seen indoors, often in hospitals or in bathrooms recording the process of shaving their hair due to the effects of chemotherapy. Nature is rarely present, and both the physical appearance and emotional expressions of the patients with cancer in these videos is in line with what one would expect of those undergoing cancer treatment: subdued or sad emotions, teary expressions, and pale skin are prevalent throughout this sample. In effect, this acts as a counterpoint to the visions of health and vitality portrayed in the alt-health sample and presents a picture to viewers and potential patients of an arduous and painful treatment and cancer journey.

Of the between 20% and 80% of patients with cancer who claim that they use alternative treatments at least supplementary to their conventional treatment, many will venture onto the web to search for resources [37,46-48]. However, patients and carers on TikTok will not just be exposed to relatively harmless advice to consider nonmedical wellness behavior such as adopting a healthy diet and avoiding harmful substances alongside chemotherapy or radiation treatment but will also encounter content advising them to replace these medically proven treatments with supposed “natural” alternatives. Beyond this, users searching for natural treatment through the search terms employed in this study will invariably come across videos that combine misinformation about the effectiveness of these supposed treatments with conspiracy ideology. Times of crisis, turmoil, and confusion can make individuals vulnerable to the influence of conspiracy ideologies—for patients navigating the throes of a cancer diagnosis, encountering content claiming that governments and pharmaceutical companies are concealing effective cancer treatments (conspiracies that are often laced with anti-Semitic ideology) could prove especially dangerous. This becomes especially concerning as we consider which narratives users are exposed to over time as opposed to in just 1 post: users do not usually make up their mind on the basis of rational arguments or even just 1 post; instead, they examine content fragments as part of a greater narrative and ideological position [112].

This study did not aim to be a comprehensive study of all visual features of TikTok videos on the topic of cancer. We selected features that were deemed theoretically relevant to the topic at hand, 3 of which (testimonials, scene and nature, and color choice) were successfully analyzable using computer vision. Further research aiming to use similar methodology on other topics will likely have to adapt their selection of visual features accordingly (although we expect testimonials to be a relevant phenomenon of much audiovisual social media content overall). Just as the examination of visual features is not exhaustive, there remain other elements of social media videos that were not considered in this study that could be of note, such as captions, soundtracks, and emojis. Future research should examine other elements of the platform’s rich audiovisual environment [113]. Finally, videos were collected via search phrases, and the TikTok app’s algorithms are opaque in their functioning. However, this method mirrors the way users would typically search for and encounter content if they were to seek information about cancer on TikTok.

TikTok’s affordances privilege communication that is emotive and personable, and these meanings are substantially transported through visual features [87]. Political campaigns have made moves to adapt to this style of communication [101,102]. Our findings demonstrate that health communication cannot rely on the truthful and rational depiction of medical facts alone and must also consider adapting to this new audiovisual platform and the norms that govern it.

### Conclusions

On topics ranging from vaccines and nutrition to wellness, the internet has become a central source of both factual and false information for patients [114]. Our examination of the public communication environment about cancer on TikTok, a central social media platform, found that different types of information are spread in different ways: compared to the conventional medicine sample videos, the alternative health sample videos rely more heavily on testimonial presentation and feature more natural elements and settings. We also add to a growing body of research on the role of color in images and videos: we found that cool-toned videos are more frequently found in the alt-health sample than in the conventional medicine sample. Our findings suggest that the alternative health community leverages the advantages of the persuasive power of audiovisual social media platforms such as TikTok. Through their visual choices and language, they portray wellness where the conventional medicine sample portrays illness, thus positing a choice to viewers between 2 unequal (supposed) solutions to their diagnosis.

Our findings also provide insights for public health scholars and practitioners: while it is important to provide patients with accurate, reliable information, including on social media platforms such as TikTok, this may not be the most successful approach or align with what users are seeking on the internet. In part, this is due to the logic of social media, which privileges all but dry, factual information. Beyond this, it is because patients scouring social media may not be rationally looking for information to inform their treatment decisions but instead searching for ways to cope with the disease in a manner that gives them hope. Alt-health influencers use the visual language of the testimonial, among other things, to build authenticity and rapport with their users and, crucially, create hope for an easier, less arduous road to recovery. Medical professionals and public health scholars might consider using similar visual language to meet patients’ emotional needs online, rather than focusing solely on their information needs.

## Abbreviations

RQ: research question

## Data Availability

The datasets generated and analyzed during this study are not publicly available to preserve the anonymity of the uploaders of the videos included in the dataset but are available from the corresponding author on reasonable request.

## References

[1] Bovet A, Makse HA (2019). Influence of fake news in Twitter during the 2016 US presidential election. Nat Commun.

[2] Grimes DR (2021). Medical disinformation and the unviable nature of COVID-19 conspiracy theories. PLoS One.

[3] Kucharski A (2016). Post-truth: study epidemiology of fake news. Nature.

[4] Suarez-Lledo V, Alvarez-Galvez J (2021). Prevalence of health misinformation on social media: systematic review. J Med Internet Res.

[5] Kitta A (2018). Alternative health websites and fake news: taking a stab at definition, genre, and belief. J Am Folk.

[6] Cuan-Baltazar JY, Muñoz-Perez MJ, Robledo-Vega C, Pérez-Zepeda MF, Soto-Vega E (2020). Misinformation of COVID-19 on the internet: infodemiology study. JMIR Public Health Surveill.

[7] (2020). Immunizing the public against misinformation. World Health Organization.

[8] Nguyen A, Catalan-Matamoros D (2020). Digital mis/disinformation and public engagement with health and science controversies: fresh perspectives from Covid-19. Media Commun.

[9] Lee JJ, Kang KA, Wang MP, Zhao SZ, Wong JY, O'Connor S, Yang SC, Shin S (2020). Associations between COVID-19 misinformation exposure and belief with COVID-19 knowledge and preventive behaviors: cross-sectional online study. J Med Internet Res.

[10] (2020). UN tackles ‘infodemic’ of misinformation and cybercrime in COVID-19 crisis. United Nations.

[11] Chen L, Wang X, Peng TQ (2018). Nature and diffusion of gynecologic cancer-related misinformation on social media: analysis of tweets. J Med Internet Res.

[12] Hernandez D, McMillan R (2019). Facebook, YouTube overrun with bogus cancer-treatment claims. The Wall Street Journal.

[13] Grimes DR (2019). How to survive the fake news about cancer. The Guardian.

[14] Wardle C (2023). Tackling misinformation: a three-pronged approach. National Institutes of Health.

[15] Dunwoody S (2020). Science journalism and pandemic uncertainty. Media Commun.

[16] Lee SY, Hawkins RP (2016). Worry as an uncertainty-associated emotion: exploring the role of worry in health information seeking. Health Commun.

[17] Liu J, King AJ, Margolin D, Niederdeppe J (2020). Information seeking and scanning about colorectal cancer screening among Black and White Americans, ages 45-74: comparing information sources and screening behaviors. J Health Commun.

[18] Gaysynsky A, Senft Everson N, Heley K, Chou WY (2024). Perceptions of health misinformation on social media: cross-sectional survey study. JMIR Infodemiology.

[19] Warner EL, Waters AR, Cloyes KG, Ellington L, Kirchhoff AC (2021). Young adult cancer caregivers' exposure to cancer misinformation on social media. Cancer.

[20] Chotipanich A, Sooksrisawat C, Jittiworapan B (2019). Association between complementary and alternative medicine use and prolonged time to conventional treatment among Thai cancer patients in a tertiary-care hospital. PeerJ.

[21] Davis GE, Bryson CL, Yueh B, McDonell MB, Micek MA, Fihn SD (2006). Treatment delay associated with alternative medicine use among veterans with head and neck cancer. Head Neck.

[22] Hanna TP, King WD, Thibodeau S, Jalink M, Paulin GA, Harvey-Jones E, O'Sullivan DE, Booth CM, Sullivan R, Aggarwal A (2020). Mortality due to cancer treatment delay: systematic review and meta-analysis. BMJ.

[23] Grimes DR (2022). The struggle against cancer misinformation. Cancer Discov.

[24] Abraham J, White KM (2017). Tracking the changing landscape of corporate wellness companies. Health Aff.

[25] Kickbusch I, Payne L (2003). Twenty-first century health promotion: the public health revolution meets the wellness revolution. Health Promot Int.

[26] Lagrosen S, Lagrosen Y, Lind L (2015). Health leadership in the wellness industry. Proceedings of the 3rd International Conference on Management, Leadership And Governance.

[27] Lofft Z (2020). When social media met nutrition. Health Sci Inq.

[28] Wellman ML (2023). “A friend who knows what they’re talking about”: extending source credibility theory to analyze the wellness influencer industry on Instagram. New Media Soc.

[29] Baker SA (2022). Alt. health influencers: how wellness culture and web culture have been weaponised to promote conspiracy theories and far-right extremism during the COVID-19 pandemic. Eur J Cult Stud.

[30] Bhargava P, MacDonald K, Newton C, Lin H, Pennycook G (2023). How effective are TikTok misinformation debunking videos?. Harv Kennedy Sch Misinformation Rev.

[31] Bursztynsky J (2021). TikTok says 1 billion people use the app each month. CNBC.

[32] Huang K (2022). For Gen Z, TikTok is the new search engine. The New York Times.

[33] Johnson SB, Parsons M, Dorff T, Moran MS, Ward JH, Cohen SA, Akerley W, Bauman J, Hubbard J, Spratt DE, Bylund CL, Swire-Thompson B, Onega T, Scherer LD, Tward J, Fagerlin A (2022). Cancer misinformation and harmful information on Facebook and other social media: a brief report. J Natl Cancer Inst.

[34] Bal R, Sinha S, Dutta S, Joshi R, Ghosh S, Dutt R (2020). Analysing the extent of misinformation in cancer related tweets. Proceedings of the International AAAI Conference on Web and Social Media.

[35] Gage-Bouchard EA, LaValley S, Warunek M, Beaupin LK, Mollica M (2018). Is cancer information exchanged on social media scientifically accurate?. J Cancer Educ.

[36] Forster K (2017). Revealed: how dangerous fake health news conquered Facebook. The Independent.

[37] (2018). ASCO 2018 cancer opinions survey. American Society of Clinical Oncology.

[38] Oliver JE, Wood T (2014). Medical conspiracy theories and health behaviors in the United States. JAMA Intern Med.

[39] Complementary and Alternative Medicine for Patients. National Cancer Institute.

[40] McKee J (1988). Holistic health and the critique of western medicine. Soc Sci Med.

[41] Sointu E (2006). The search for wellbeing in alternative and complementary health practices. Sociol Health Illn.

[42] Complementary and alternative medicine. National Institutes of Health National Cancer Institute.

[43] Gerson therapy (PDQ®)–patient version. National Institutes of Health National Cancer Institute.

[44] Callaghan S, Lösch M, Pione A, Teichner W (2021). Feeling good: the future of the $1.5 trillion wellness market. McKinsey & Company.

[45] Eisenberg DM, Davis RB, Ettner SL, Appel S, Wilkey S, Van Rompay M, Kessler RC (1998). Trends in alternative medicine use in the United States, 1990-1997: results of a follow-up national survey. JAMA.

[46] Alsharif F (2021). Discovering the use of complementary and alternative medicine in oncology patients: a systematic literature review. Evid Based Complement Alternat Med.

[47] Johnson SB, Park HS, Gross CP, Yu JB (2018). Complementary medicine, refusal of conventional cancer therapy, and survival among patients with curable cancers. JAMA Oncol.

[48] (2022). Dietary supplements: underregulated, unknown and maybe unsafe. American Medical Association.

[49] Maritess C, Small S, Waltz-Hill M (2005). Alternative nutrition therapies in cancer patients. Semin Oncol Nurs.

[50] Newman M (2018). Is cancer fundraising fuelling quackery?. BMJ.

[51] Zenone M, Snyder J, Bélisle-Pipon JC, Caulfield T, van Schalkwyk M, Maani N (2023). Advertising alternative cancer treatments and approaches on meta social media platforms: content analysis. JMIR Infodemiology.

[52] Carrion-Alvarez D, Tijerina-Salina PX (2020). Fake news in COVID-19: a perspective. Health Promot Perspect.

[53] Peng W, Lim S, Meng J (2022). Persuasive strategies in online health misinformation: a systematic review. Inf Commun Soc.

[54] Blaskiewicz R (2013). The Big Pharma conspiracy theory. Med Writing.

[55] Derkatch C (2018). The self-generating language of wellness and natural health. Rhetoric Health Med.

[56] The Lancet Oncology (2018). Oncology, "fake" news, and legal liability. Lancet Oncol.

[57] Nguyen A, Vu HT (2019). Testing popular news discourse on the “echo chamber” effect: does political polarisation occur among those relying on social media as their primary politics news source?. First Monday.

[58] Warren KE, Wen LS (2017). Measles, social media and surveillance in Baltimore City. J Public Health.

[59] Lee MT, Theokary C (2021). The superstar social media influencer: exploiting linguistic style and emotional contagion over content?. J Bus Res.

[60] Moyer-Gusé E (2008). Toward a theory of entertainment persuasion: explaining the persuasive effects of entertainment-education messages. Commun Theory.

[61] Fisher WR (2009). Narration as a human communication paradigm: the case of public moral argument. Commun Monogr.

[62] Margolin DB (2020). The theory of informative fictions: a character-based approach to false news and other misinformation. Commun Theory.

[63] Hamby A, Daniloski K, Brinberg D (2015). How consumer reviews persuade through narratives. J Bus Res.

[64] Fisher WR (2009). The narrative paradigm: an elaboration. Commun Monogr.

[65] Mar RA, Oatley K, Djikic M, Mullin J (2011). Emotion and narrative fiction: interactive influences before, during, and after reading. Cogn Emot.

[66] Breil C, Böckler A (2021). Look away to listen: the interplay of emotional context and eye contact in video conversations. Visual Cognition.

[67] Burgoon JK, Birk T, Pfau M (1990). Nonverbal behaviors, persuasion, and credibility. Human Comm Res.

[68] Lim H, Childs M (2020). Visual storytelling on Instagram: branded photo narrative and the role of telepresence. J Res Interact Market.

[69] Pera R, Viglia G (2016). Exploring how video digital storytelling builds relationship experiences. Psychol Mark.

[70] Grenard JL, Uy V, Pagán JA, Frosch DL (2011). Seniors' perceptions of prescription drug advertisements: a pilot study of the potential impact on informed decision making. Patient Educ Couns.

[71] Appiah O (2006). Rich media, poor media: the impact of audio/video vs. text/picture testimonial ads on browsers' evaluations of commercial web sites and online products. J Curr Issues Res Advert.

[72] de Wit JB, Das E, Vet R (2008). What works best: objective statistics or a personal testimonial? An assessment of the persuasive effects of different types of message evidence on risk perception. Health Psychol.

[73] Martin BA, Wentzel D, Tomczak T (2008). Effects of susceptibility to normative influence and type of testimonial on attitudes toward print advertising. J Advert.

[74] Morton H (2017). The new visual testimonial: narrative, authenticity, and subjectivity in emerging commercial photographic practice. Multidiscip Stud Media Commun.

[75] Donelly B, Toscano N (2017). The Woman Who Fooled The World: Belle Gibson's Cancer Con, and the Darkness at the Heart of the Wellness Industry.

[76] Bainotti L, Caliandro A, Gandini A (2020). From archive cultures to ephemeral content, and back: studying Instagram stories with digital methods. New Media Soc.

[77] Page R, De Fina A, Georgakopoulou A (2015). The narrative dimensions of social media storytelling: options for linearity and tellership. The Handbook of Narrative Analysis.

[78] Klein EJ, Berube DM (2021). Fake news about Covid 19: communication strategies on WhatsApp in Brazil. Pandemic Communication and Resilience.

[79] Venditti S, Piredda F, Mattana W (2017). Micronarratives as the form of contemporary communication. Design J.

[80] Eisenlauer VJ, Smith B, O'Halloran K (2011). Multimodality and social actions in 'personal publishing' text: from the German 'poetry album' to web 2.0 'social network sites. Multimodal Studies: Exploring Issues and Domains.

[81] Gibson JJ (1979). The Ecological Approach to Visual Perception: Classic Edition.

[82] Struik LL, Bottorff JL, Baskerville NB, Oliffe JL (2018). The Crush the Crave quit smoking app and young adult smokers: qualitative case study of affordances. JMIR Mhealth Uhealth.

[83] Moreno MA, D'Angelo J (2019). Social media intervention design: applying an affordances framework. J Med Internet Res.

[84] Chong I, Proctor RW (2020). On the evolution of a radical concept: affordances according to Gibson and their subsequent use and development. Perspect Psychol Sci.

[85] boyd d (2010). Social network sites as networked publics: affordances, dynamics, and implications. A Networked Self.

[86] Schellewald A (2023). Understanding the popularity and affordances of TikTok through user experiences. Media Culture Soc.

[87] Zhao H, Wagner C (2022). How TikTok leads users to flow experience: investigating the effects of technology affordances with user experience level and video length as moderators. Internet Res.

[88] Lu Y, (Cindy) Shen C (2023). Unpacking multimodal fact-checking: features and engagement of fact-checking videos on Chinese TikTok (Douyin). Soc Media Society.

[89] Peng Y, Jemmott JB III (2018). Feast for the eyes: effects of food perceptions and computer vision features on food photo popularity. Int J Commun.

[90] Kizilcec RF, Papadopoulos K, Sritanyaratana L (2014). Showing face in video instruction: effects on information retention, visual attention, and affect. Proceedings of the SIGCHI Conference on Human Factors in Computing Systems.

[91] Li Y, Xie Y (2019). Is a picture worth a thousand words? An empirical study of image content and social media engagement. J Mark Res.

[92] Goren CC, Sarty M, Wu PY (1975). Visual following and pattern discrimination of face-like stimuli by newborn infants. Pediatrics.

[93] Johnson CI, Mayer RE (2012). An eye movement analysis of the spatial contiguity effect in multimedia learning. J Exp Psychol Appl.

[94] Teixeira T, Wedel M, Pieters R (2012). Emotion-induced engagement in internet video advertisements. J Mark Res.

[95] Ostrovsky AM, Chen JR (2020). TikTok and its role in COVID-19 information propagation. J Adolesc Health.

[96] Kong W, Song S, Zhao YC, Zhu Q, Sha L (2021). TikTok as a health information source: assessment of the quality of information in diabetes-related videos. J Med Internet Res.

[97] Song S, Xue X, Zhao YC, Li J, Zhu Q, Zhao M (2021). Short-video apps as a health information source for chronic obstructive pulmonary disease: information quality assessment of TikTok videos. J Med Internet Res.

[98] García CA, Duncan P, Goodier M, Hunter-Green Z (2024). #ukpolitics: how the 2024 general election has played out on TikTok. The Guardian.

[99] Ward J (2024). Donald Trump joins TikTok and rapidly wins three million followers. Reuters.

[100] Gilbert D (2024). TikTok pushed young german voters toward far-right party. Wired.

[101] Cervi L, Tejedor S, Blesa FG (2023). TikTok and political communication: the latest frontier of politainment? A case study. Media Commun.

[102] Bösch M (2021). Broken promises: TikTok and the German election. The Mozilla Foundation.

[103] Serrano JC, Papakyriakopoulos O, Hegelich S (2020). Dancing to the partisan beat: a first analysis of political communication on TikTok. Proceedings of the 12th ACM Conference on Web Science.

[104] Joo J, Steinert-Threlkeld ZC Image as data: automated visual content analysis for political science. arXiv.

[105] Geitgey A (2021). ageitgey / face_recognition. GitHub.

[106] Zhou B (2017). CSAILVision / places365. GitHub.

[107] Zhou B, Lapedriza A, Khosla A, Torralba A, Oliva A (2017). Places. Massachusetts Institute of Technology.

[108] Emorine B (2020). Boris-Em / ColorKit. GitHub.

[109] Braun V, Clarke V (2006). Using thematic analysis in psychology. Qual Res Psychol.

[110] Wellman ML, Holton AE, Kaphingst KA (2023). Previvorship posting: why breast cancer previvors share their stories on social media. Health Commun.

[111] Milani E, Weitkamp E, Webb P (2020). The visual vaccine debate on Twitter: a social network analysis. Media Commun.

[112] Wardle C (2023). Misunderstanding misinformation. Issues Sci Technol.

[113] Fornara F, Lomicka LL (2019). Using visual social media in language learning to investigate the role of social presence. CALICO J.

[114] Skafle I, Nordahl-Hansen A, Quintana DS, Wynn R, Gabarron E (2022). Misinformation about COVID-19 vaccines on social media: rapid review. J Med Internet Res.

